# Pathophysiology of pain in acute pancreatitis: A narrative review

**DOI:** 10.1177/20494637261496489

**Published:** 2026-10-14

**Authors:** Patrice Forget, Urszula Brzuchalska, Sören Marsmann, Sara Brophy, Brittany Chimsuk, Brooke Irvine, Conor Logan, Sophie Macdonald, Andrew Mands, Sharon Vivienne Mathews, Mark Mcmillan, Sneha Prothasis, Emma Thomson, Asbjørn Mohr Drewes, Sanjay Pandanaboyana, Esther Pogatzki-Zahn

**Affiliations:** 1 Institute of Applied Health Sciences, School of Medicine, University of Aberdeen, Aberdeen, UK; 2 Department of Anesthesia, NHS Grampian, Aberdeen, UK; 3 School of Medicine, 1019 University of Aberdeen, Aberdeen, UK; 4 Department of Anaesthesiology, Intensive Care and Pain Medicine, University Hospital Muenster, Muenster, Germany; 5 Department of Gastroenterology & Hepatology, Aalborg University Hospital, Aalborg, Denmark; 6 Department of HPB and Transplant Surgery, 105565 Freeman Hospital, Newcastle Upon Tyne, UK

**Keywords:** pain, acute pain, nociceptors, nociceptive pain, visceral pain, pain-referred

## Abstract

**Background and objective:**

Pain affects between 80 and 95% of patients with acute pancreatitis (AP). However, despite its importance and severity, the pathophysiological mechanisms are not well understood. This narrative review aims to review current knowledge about the pathophysiological mechanisms of pain in AP.

**Methods, databases and data treatment:**

A search of electronic databases (PubMed/MEDLINE, Embase, and Cochrane) was conducted from January 31 2024 to July 25 2025. Inclusion criteria included any study on acute pancreatitis and pain (management). Exclusion criteria included the absence of relevant content or specific information about pain in AP.

**Results:**

We identified 11 studies. Two potential pain pathways were identified, categorised as nociceptive and neuropathic. Nociceptive signals are enhanced through the release of inflammatory mediators, such as nuclear factor-kappa B, nitric oxide and interleukin-6, in response to tissue damage. Furthermore, the characteristic visceral and referred pain is likely attributed to high levels of overlap of primary sensory afferent fibre activation and neuronal convergence. Damage of the central and peripheral nervous system through the upregulation of voltage-gated ion channels, vanilloid receptors and transmembrane proteins may cause neuropathic-like pain. This triggers neurogenic inflammation and further release of neuropeptides, inducing an auto-amplification loop between neurogenic inflammation and neuropathic pain, with one positively reinforcing the other.

**Conclusions:**

Diagnostic approaches in AP should consider nociceptive and neuropathic mechanisms, which should guide specific pain management. However, the knowledge gap remains significant, for example, for specific biomarkers and mechanisms enabling the development of target-specific personalised treatments for pain in AP in the future.

**Significance statement:**

In 2023, the James Lind Alliance priority-setting partnership in pancreatitis indicated that the number one priority for AP is establishing more effective ways to manage pain.^1^ Therefore, a complete comprehensive understanding of the pathophysiology of pain in AP should be obtained, to advise specific pain management guidelines in AP. This review is the first of its kind, attempting to synthesise the collective knowledge on pathophysiology of pain in AP.

## Introduction

Acute pancreatitis (AP) is described as the sudden onset of inflammation of the pancreas, characterised by severe epigastric pain that may radiate to the back.^2^ The symptoms of AP include vomiting, nausea, fever and pain (diffuse or epigastric, which afflicts 80-95% of patients).^3^ AP is a highly prevalent global condition with increasing incidence.^4^ Considering that pain is prevalent in the majority of cases, this can be thought of as a major problem considering that the pain associated with AP affects a large population worldwide.^3^ The pain intensity is typically high, warranting intense treatment. Hence, a study which used the Visual Analog Scale to assess pain in AP and place it on a continuum from 1 (minimal pain) to 10 (agonising pain) showed that 70.3% of participants rated their pain within the 7-10 range.^5^

The pancreas itself is a retroperitoneal organ, found on the posterior wall of the upper abdomen.^6,7^ It is a highly innervated organ that has strong associations with both the enteric and central nervous systems which is vital to complete its functions of assisting with macronutrient digestion and blood glucose homeostasis.^7,8^

Common causes of AP include gallstones, excessive alcohol intake and hyperlipidaemia.^2^ Gallstone-derived AP is twice as common as AP associated with alcohol misuse and is thought to be associated with a rising level of obesity worldwide.^9^ This occurs most commonly in microlithiasis patients, due to pancreatic ampullary obstruction. Consequential reflux of pancreatic secretions in turn gives rise to inflammation and pancreatic damage.^10^ Other causes of AP include endoscopic retrograde cholangiopancreatography (ERCP), hypertriglyceridemia, and hypercalcaemia.^11^ It may also occur without discernible cause, deemed idiopathic in 15% of cases.^11^ The multiple pathophysiological mechanisms underlying this condition are inadequately understood, and pain management may vary between the conditions.^2^

The Atlanta classification (revised in 2011) is widely used to diagnose and classify AP.^2^ Current NICE Guidelines on the management of AP only give recommendations on general disease management.^12^ Importantly, these guidelines lack recommendations for the management of pain in AP. This is a similar trend across many AP international guidelines.^13^ Despite the recommendations for general disease management, an absence of any clear guidelines regarding pain control is a significant concern (however new guidelines supported by United European Gastroenterology is expected in the fall of 2025), particularly with recent emerging data suggesting high opioid use for pain management in AP.^14^ There appears to be limited emphasis in research on the associated pain. One basis for treating pain appropriately is identifying the underlying mechanisms of pain related to a particular disease. Only if the pathophysiology that is responsible for pain in AP is identified, adequate treatment options can be determined and new, specific treatments can be developed.

The present manuscript aims to review the pathophysiology behind pain pathways in AP including a detailed exploration of the connection between nociceptive and neuropathic pain in AP, with the aim to help inform future guidelines regarding analgesic strategy.

## Methods

A search of electronic databases (PubMed/MEDLINE, Embase, and Cochrane) was conducted with keywords ‘*acute pancreatitis’, ‘pain’, ‘nociceptive pain’, ‘neuropathic pain’ *and* ‘acute pancreatitis management’. *Literature included in this review was (1) published between January 31 2024 to July 25 2025 and (2) full text available in English. Papers that featured the absence of relevant content or specific information about pain in AP were excluded. Evidence from these papers was extracted, qualitatively analysed and summarised.

Screening was performed by three authors (BI, SB, BCSP) and included publications were decided upon by author discretion based on the relevancy to this review’s aim. Data were manually extracted, discussed, classified and synthesised. Additional studies were considered to recontextualize research findings alongside the reference lists of the included papers being reviewed.

## Results

From 379 studies identified on PubMed/MEDLINE, Embase, and Cochrane, 11 were included based on the inclusion criteria. Six were excluded based on the exclusion criteria and six others were identified through reference lists. We finally included 11 studies, of which 8 included preclinical,^2,15–21^ and 4 clinical data^2,5,22,23^. Based on the results of these studies, the following overview can be given to understand the pathophysiology of pain related to AP, based on current knowledge.

### General pathophysiology of pain related to AP

Severe epigastric pain is a significant symptom of AP and despite its prevalence, the management of this pain remains controversial, highlighting the incomplete understanding of the underlying pain mechanisms.^16^ Understanding the pathophysiology of pain in AP first requires consideration of normal pancreatic enzyme physiology, followed by the pathological processes that occur during pancreatic inflammation and injury.

Under normal physiological conditions, pancreatic acinar cells synthesise digestive enzyme precursors, including trypsinogen, which are secreted into the duodenum. Within the duodenal mucosa, enterokinase produced by the crypts of Lieberkühn converts trypsinogen into active trypsin.^24^ This spatial separation between pancreatic enzyme production and activation protects the pancreas from autodigestion. In addition, pancreatic acinar cells utilise intracellular degradation pathways, including autophagy, to recycle damaged cellular components.^25^

In AP, this protective separation of enzyme synthesis and activation becomes disrupted. Although the precise mechanisms remain misunderstood, premature intracellular activation of trypsinogen within pancreatic acinar cells is considered a key event in disease initiation.^26^ Experimental evidence suggests that abnormal co-localisation of zymogen granules and lysosomal compartments may contribute to this process, as lysosomal hydrolases can activate trypsinogen within the pancreas.^26^ However, the roles of autophagy and lysosomal dysfunction in AP remain complex and are still being investigated.^25^ Premature activation of trypsin subsequently leads to autolytic injury of pancreatic tissue and promotes activation of downstream digestive enzymes, resulting in acinar cell injury and necrosis.^26^

The resulting tissue injury initiates a profound inflammatory response characterised by neutrophil recruitment and local leucocyte activation.^27^ Increased capillary permeability allows cytokines, chemokines, and other inflammatory mediators released from injured acinar cells to enter the systemic circulation, potentially leading to a systemic inflammatory response syndrome (SIRS).^22^ The systemic consequences of AP extend beyond the pancreas and may ultimately result in multi-organ dysfunction, with approximately 20% of patients developing some form of non-pancreatic organ failure.^22,28^

The inflammatory and necrotic processes of AP are closely associated with the generation of pain. In general, nociception refers to the physiological process by which the nervous system detects and encodes potentially harmful stimuli.^29^ This process underlies the perception of pain, defined as ‘An unpleasant sensory and emotional experience associated with, or resembling that associated with, actual or potential tissue damage’.^30^ The pancreas is innervated by a complex network of afferent nerve fibres. Parasympathetic fibres arise from the abdominal vagus nerve, while sympathetic afferents travel via the greater and lesser splanchnic nerves.^16^ The sympathetic pathways are particularly associated with the visceral pain experienced in AP.^16^ Additionally, the enteric nervous system (ENS) may contribute to neurogenic reflex pain through mechanisms seen in dysmotility and ileus.^31^

Pancreatic nociception begins with activation of nociceptors located at the terminals of primary afferent neurons.^29^ Noxious mechanical, thermal, and chemical stimuli generated during pancreatic inflammation and tissue injury activate these nociceptors, producing depolarisation and the generation of action potentials that are transmitted to the spinal cord.^29^ Nociceptors may consist of myelinated Aδ fibres or unmyelinated C fibres.^32^ Aδ fibres typically mediate sharp, well-localised pain, whereas C fibres mediate the diffuse, dull, and poorly localised visceral pain more characteristic of AP.^32^ As the pancreas is a visceral organ, pain is predominantly mediated by C fibres of the autonomic nervous system. However, involvement of the parietal peritoneum may recruit somatic afferents and produce more localised pain mediated by Aδ fibres.

Beyond direct nociceptor activation, pancreatic inflammation also promotes peripheral sensitisation. Damaged acinar cells, infiltrating immune cells, and injured neural tissue release pro-inflammatory mediators that lower nociceptor activation thresholds. This increases neuronal responsiveness to subsequent stimulation.^32^ In addition, ‘silent’ nociceptors within the pancreas may become activated in the presence of inflammatory mediators, further amplifying pain signalling during AP.^32^

### Nociceptive pain in AP

Pain mechanisms are complex with multiple pathways working cooperatively. Whether through nociceptive pain via either the somatic or visceral pain pathway or neuropathic pain as a consequence of nerve damage.^15^

As aforementioned, nociceptors serve an important role in the body; for example, in the somatic system their mechanism acts to protect the body from harm.^29^ In this process inflammatory mediators are released due to inflammation or tissue damage.^2^ Nuclear Factor Kappa B (NF-κB) is stored within the cytoplasm in an inactive state along with its inhibitory proteins known as IκBs. Both play a crucial rolein the transcriptionof genes involved in regulating numerous aspects of the immune system. NF-κB begins to transcribe increasing amounts of pro-inflammatory mediators in response to acinar cell injury.^2^ Overactivation of the NF-κB pathway may subsequently contribute to nociceptor sensitisation and amplification of inflammation at both cellular and systemic levels.^2^ Clinically, this may manifest as systemic inflammatory response syndrome (SIRS), multiple organ dysfunction syndrome (MODS) and acute respiratory distress syndrome (ARDS).^33^

Nitric oxide (NO) plays a significant role as a signalling molecule in the pathophysiology of inflammation and pain induction. A study on the correlation between NO and AP demonstrated increased NO production following the onset of AP.^34^ Elevated NO levels may further enhance activation of the NF-κB cascade, contributing to inflammatory signalling and sensitisation of nociceptive pathways.^34^

NO signalling has additionally been implicated in chronic and neuropathic pain through the modulation of peripheral nociceptors and central sensitisation mechanism.^35,36^ However, despite mechanistic overlap between NO-mediated signalling, pain generation and AP severity, the discriminative ability of severe pain for predicting AP outcomes appears limited.

A recent international prospective study demonstrated that although severe pain was associated with moderately severe/severe AP, pancreatic necrosis, and renal organ failure, its overall predictive performance for AP outcomes remained poor.^37^ This discrepancy may reflect the multifactorial nature of AP progression, whereby inflammatory, neuroimmune and biochemical pathways associated with disease severity are not fully captured by subjective pain perception alone.^21,37^ Pain intensity is also influenced by individual variation in nociceptive processing, psychological factors, analgesic exposure and timing of assessment.^38,39^ Consequently, although NO-mediated pathways may contribute to both pain generation and disease severity,^34^ pain severity itself may not serve as a reliable marker for adverse AP outcomes.^37^

Apart from the somatic inflammatory response, visceral pain is also a subtype of nociceptive pain. Collett (2013) outlines visceral pain as: ‘the pain we feel when our internal organs are inflamed, diseased, damaged or injured’.^40^ The gut-brain axis forms a two-way communication system between the nervous system and gastrointestinal tract – allowing mechanical, chemical and micro-bacterial stimuli in the gut to trigger sensory neurons to peripherally sensitise and onset the perception of visceral pain.^41^ Visceral pain from the pancreas is generally not well-localised. It is transmitted by nociceptive afferent fibres with contribution from the vagus nerve – which have a terminating pattern on the spinal cord that is diffuse and wide. This may be why the pain in AP is often generalised and difficult to localise, being described as dull although severe.^27,42^

To be more specific, the pancreatic nociceptive afferents lead to spinal cord level T5-L2.^2^ From there, the neurons synapse at the level of entry then travels up through the spinothalamic tract to the thalamus or alternatively through the spinoreticulothalamic pathway before being projected to other areas of the brain such as the somatosensory cortex for pain processing.^43^ Preventing nerve transmission along these fibres can help stop the transfer of nociceptive pain from AP. This is the basis of epidural analgesia – providing a nerve block stopping sensory information being transmitted.^44^

Pancreatic afferent nerves connect with the celiac ganglions of this plexus, so similarly, blocking the ganglion results in blocking nociceptive signals leading to reduced pain sensation.^45^ Concerning celiac plexus blocks, the research on pancreatic cancer and chronic pancreatitis is far more extensive than that of acute pancreatitis. Therefore, its place in AP pain management is yet to be shown^35,46,47,^.

Another feature of pain sensation in AP is referred pain. Referred pain is pain felt adjacent to or even at a distance from the site of origin of the pain.^48^ In AP, pain can often be felt in the left upper quadrant, right upper quadrant or radiate to the back.^42^ This could be due to high levels of sensory overlap in the abdominal viscera, with nerve fibres from the same spinal cord segment innervating multiple different organs (cross-organ sensitisation).^49^ The origin of this is embryonic, with nerve supplies being pulled in all directions, following the growth and rotation of the abdominal organs.^50^ The brain has the ability to perceive pain coming from a specific spinal segment but not from which exact afferents. Therefore, AP pain may be felt in different regions, differing in presentation from person to person.

The literature on AP has also mentioned visceral hypersensitivity as a contributor to pain sensation during pancreatitis.^2,51^ As pain intensity increases, so do the expressions of these factors and proteins. Nevertheless, it is essential to note that the referenced studies also include patients with CP. While AP and CP are closely linked, exploring the same results without specific studies on AP remains uncertain. To summarise, visceral hypersensitivity has been mentioned as a potential factor in developing pain during pancreatitis. Yet, since there is no simple explanation of how it precisely affects pain sensation in AP, and the exact mechanisms remain unclear, further studies are required to delve into this deeper.

### Neuropathic pain in AP

Neuropathic pain is described as an abnormal response to non-painful stimuli affecting the somatosensory system. Depending on the nature of the stimulus (either dynamic or constant), neuropathic pain can be described using terms such as burning, shooting or, sharp but only serve as helpful descriptors and can not be relied on to distinguish pain as exclusively neuropathic. Although evidence supports the connection between the pathogenesis of CP and neuropathic pain, direct evidence linking it with AP is currently lacking.^19,38,39^

In peripheral sensitisation, nerve lesions cause changes to uninjured primary afferent nociceptors.^2^ Factors involved in this mechanism include the expression of voltage-gated sodium channels, vanilloid receptors and other transmembrane proteins. The vanilloid transmembrane protein in hot peppers (capsaicin) has been linked to triggering neurogenic inflammation, which can result in peripheral neuropathic pain.^18^ In neurogenic inflammation, substances such as bradykinin, arachidonic acid, and hydrogen ions are released from the primary sensory nerve terminals residing in the dorsal root, triggering dorsal root reflexes. This pathological response, in turn, triggers the primary nerve terminals to induce inflammation at the target tissues through the release of neuropeptides, specifically calcitonin-gene-related peptide (CGRP) and tachykinins, namely substance P. The release of substance P catalyses a response which consequently leads to vasodilation and oedema of the pancreas and is characteristic of local inflammation. The release of substance P also directly signals the spinal cord to induce pain.^17^ The pain response is initiated secondary to the inflammation whereby the inflammation induces excitability at the spinal cord and as a result, peripheral pain signals become amplified. Neurogenic inflammation may induce an auto-amplification loop between inflammation and pain which can lead to the progression of AP^20,21^. It should be noted that neurogenic inflammation is not the only mechanism through which neuropathic pain is caused. Furthermore, it is not understood which specific mechanism contributes to the generation of pain in AP.^36^

Central sensitisation encompasses an abnormal and heightened response to stimuli that is normally non-painful in nature.^2^ In the central pathway, several factors are involved in stimulation of pain, including growth factors, pro-inflammatory cytokines and changes in ion channels. Földi et al. reported that patients with AP who described their pain as ‘sharp’ had a higher burden of disease where prognosis was worse, and mortality rates were higher.^5^ While ‘sharp’ pain has been suggested to be neuropathic, this descriptor is not specific and current evidence is insufficient to conclude this relation. Nevertheless, as an association between pain descriptors and disease severity is reported, incorporating qualitative assessment of pain may have potential prognostic value in patients with AP but warrants further research.

## Discussion

Overall, this paper summarises the AP specific inflammatory processes and finds nociceptive pain to be well documented. Studies regarding neuropathic and sensitisation of pain pathways are primarily centred around CP and often no direct link to AP is addressed. However, good arguments allow us to consider that nociceptive pain may only be one of the mechanisms involved in AP pain.

The role of cytokines in neurogenic inflammation is complex, having both pro-inflammatory and anti-inflammatory effects. Cytokine numbers rapidly increase in peripheral injury, resulting in a cellular cascade dependent on the activation of the target cell. The large number of factors and molecules means it can be difficult to target one specific molecule. It appears as an unlikely area of therapeutic targeting, although relevant to neuroinflammation.^52^ Whilst it is hypothesised that neuropathic pain may occur secondary to inflammation of afferent nociceptors in the pancreas, a lack of specific pathogenic features and availability of diagnostic pain tools severely hinders the identification of neuropathic pain in AP. This creates further issues regarding precision when interpreting complex pain descriptors and the lack of validation of pain history questionnaires.^53^ Once patients receive analgesia, specific pain reporting may become ineffective, leading to further difficulty in the quantification of pain in AP.

A third distinct type of pain, known as nociplastic pain, is potentially the most elusive. This produces a much more widespread and multifocal pain, associated with ongoing inflammation and tissue damage.^54^ We found no direct association with AP however, as the disease may progress to a more chronic picture, nociplastic pain is much more likely to play a role in the pathophysiology of pain. It is clinically important, presenting its own challenges regarding the lack of effective treatment. The constant pain present in CP is linked to poorer outcomes, potentially corresponding to nociplastic mechanisms.^55^

The recent evidence-based review by van Zeggeren et al.^56^ provides a comprehensive treatment of pain mechanisms and interventional management in CP. The present review addresses a distinct and complementary gap: the pathophysiology of pain specific to acute pancreatitis (AP). Unlike CP, where established treatment algorithms now exist, AP pain mechanisms remain an emerging area of study. Key pathways discussed here – including acute NF-κB and nitric oxide signalling, activation of silent nociceptors by inflammatory mediators, and the prognostic value of pain quality at presentation – are unique to the acute disease episode and are not captured in CP-focused literature. The clinical importance of this distinction is reinforced by the James Lind Alliance priority-setting partnership, which identified pain management in AP as the leading research priority in pancreatitis research in 2023.^1^

Despite our extensive examination of the available literature, it is possible that certain areas of the topic may not be thoroughly explored or included in our review. Additionally, the scope of our review means that we may have excluded certain areas of current research which are yet to be published.

## Conclusion

This paper has delved into the pain mechanisms of AP in an attempt to close the gap between its unique pathophysiology related to pain and – based on this – adequate pain management strategies. The analysis of current literature and research findings underscores the complexity of AP, emphasising the need for further exploration to enhance our understanding of AP pain. Due to the complex pathophysiology, pharmacological options should be considered based on the symptoms (e.g. nociceptive versus neuropathic) and based on the severity of symptoms. Both will guide treatment and future research to investigate specific treatment options: Manifestation of pain in this context imposes significant distress, emphasising the imperative for prioritised management of AP. Implementing a patient-centred care model, tailoring analgesia to address specific pathophysiological mechanisms and severity, has the potential to yield therapeutic benefits for each individual patient.

## Appendix

### Abbreviations

AP: Acute Pancreatitis
CP: Chronic Pancreatitis
NICE: National Institute for Health and Care Excellence
NSAIDs: Non-Steroidal Anti-Inflammatory Drugs
UK: United Kingdom.

## References

[1] Mitra V Munnelly S Grammatikopoulos T , et al. The top 10 research priorities for pancreatitis: findings from a James Lind alliance priority setting partnership. The Lancet Gastroenterology & hepatology 2023; 8(9):780–782. 10.1016/s2468-1253(23)00151-6, Available from: https://www.thelancet.com/journals/langas/article/PIIS2468-1253(23)00151-6/abstract 37356452

[2] Beiriger J Khan A Yan B , et al. Comprehensive review of acute pancreatitis pain syndrome. Gastrointestinal disorders 2023; 5(2): 144–166. 10.3390/gidisord5020014

[3] Szatmary P Grammatikopoulos T Cai W , et al. Acute pancreatitis: diagnosis and treatment. Drugs 2022; 82(12): 1251–1276. 10.1007/s40265-022-01766-4 PMC9454414 36074322

[4] Matta B Gougol A Gao X , et al. Worldwide variations in demographics, management, and outcomes of acute pancreatitis. Clin Gastroenterol Hepatol 2020; 18(7): 1567–1575.e2. 10.1016/j.cgh.2019.11.017 PMC9198955 31712075

[5] Földi M Gede N Kiss S , et al. The characteristics and prognostic role of acute abdominal on-admission pain in acute pancreatitis: a prospective cohort analysis of 1432 cases. Eur J Pain 2021; 26(3): 610–623. 10.1002/ejp.1885 PMC9299627 34758174

[6] Mahadevan V . Anatomy of the pancreas and spleen. Surgery 2019; 37(6): 297–301, Available from: https://www.sciencedirect.com/science/article/pii/S026393191930095X/text/The/20pancreas/20is/20a/20large

[7] Walkowska J Zielinska N Karauda P , et al. The pancreas and known factors of acute pancreatitis. J Clin Med 2022; 11(19): 5565. 10.3390/jcm11195565, Available from: https://www.mdpi.com/2077-0383/11/19/5565/htm PMC9571992 36233433

[8] Nicoletti A Vitale F Paratore M , et al. Neuropancreatology: the nervous system and pain management in pancreatic diseases. Life 2024; 14(3): 299. 10.3390/life14030299, Available from: https://www.mdpi.com/2075-1729/14/3/299 PMC10970869 38541624

[9] Nesvaderani M Eslick GD Vagg D , et al. Epidemiology, aetiology and outcomes of acute pancreatitis: a retrospective cohort study. Int J Surg 2015; 23: 68–74. 10.1016/j.ijsu.2015.07.701, Available from: https://reader.elsevier.com/reader/sd/pii/S1743919115010730/token/3DFB6DA85596EA9606FBE0811B4F33E657145756113C60942592A2D2ACD2F21D26B8D2D78218C06798988E4396A2A06B 26384834

[10] Shah A Mourad M Bramhall S . Acute pancreatitis: current perspectives on diagnosis and management. Journal of Inflammation Research 2018; 11(11): 77–85. 10.2147/JIR.S135751, Available from: https://www.ncbi.nlm.nih.gov/pmc/articles/PMC5849938/PMC5849938 29563826

[11] Cappell MS. Acute pancreatitis: etiology, clinical presentation, diagnosis, and therapy. Med Clin 2008; 92(4): 889–923. 10.1016/j.mcna.2008.04.013, Available from: https://www.ubccriticalcaremedicine.ca/academic/jc_article/Acute/20Pancreatitis/20Review/20Cappel/20Med/20Clin/20North/20Am/202008/20(Jan-08-09).pdf 18570947

[12] NICE . Recommendations | pancreatitis | guidance. NICE. Nice.org.uk. NICE, 2018. Available from: https://www.nice.org.uk/guidance/ng104/chapter/Recommendations#acute-pancreatitis

[13] Cai W Liu F Wen Y , et al. Pain management in acute pancreatitis: a systematic review and meta-analysis of randomised controlled trials. Front Med 2021; 8(8): 782151. 10.3389/fmed.2021.782151 PMC8718672 34977084

[14] Knoph CS Lucocq J Kamarajah SK , et al. Global trends in opioid use for pain management in acute pancreatitis: a multicentre prospective observational study. United Eur Gastroenterol J 2024; 12(8): 1114–1127. 10.1002/ueg2.12641 PMC11485295 39140779

[15] Armstrong SA Herr MJ . Physiology, nociception. StatPearls Publishing, 2023. Available from: https://www.ncbi.nlm.nih.gov/books/NBK551562/31855389

[16] Barreto SG Saccone GTP . Pancreatic nociception – revisiting the physiology and pathophysiology. Pancreatology 2012; 12(2): 104–112. 10.1016/j.pan.2012.02.010 22487519

[17] Liddle RA Nathan JD . Neurogenic inflammation and pancreatitis. Pancreatology 2004; 4(6): 551–560. 10.1159/000082180 15550764

[18] Liddle RA . The role of transient receptor potential vanilloid 1 (TRPV1) channels in pancreatitis. Biochim Biophys Acta Mol Basis Dis 2007; 1772(8): 869–878. 10.1016/j.bbadis.2007.02.012 PMC1995747 17428642

[19] Long Y Jiang Z Wu G . Pain and its management in severe acute pancreatitis. Journal of Translational Critical Care Medicine 2022; 4(1): 9. 10.4103/jtccm-d-21-00026

[20] Vera-Portocarrero L Westlund KN . Role of neurogenic inflammation in pancreatitis and pancreatic pain. Neurosignals 2005; 14(4): 158–165. 10.1159/000087654 PMC2766588 16215298

[21] Wu Y Han C Luo R , et al. Molecular mechanisms of pain in acute pancreatitis: recent basic research advances and therapeutic implications. Front Mol Neurosci. 2023; 16: 1331438. 10.3389/fnmol.2023.1331438 PMC10771850 38188196

[22] Garg PK Singh VP . Organ failure due to systemic injury in acute pancreatitis. Gastroenterology 2019; 156(7): 2008–2023. 10.1053/j.gastro.2018.12.041, Available from: https://www.ncbi.nlm.nih.gov/pmc/articles/PMC6486861/PMC6486861 30768987

[23] Cruciani RA Jain S . Pancreatic pain: a mini review. Pancreatology 2008; 8(3): 230–235. 10.1159/000134278 18509251

[24] Jones MR Hall OM Kaye AM , et al. Drug-induced acute pancreatitis: a review. Ochsner J 2015; 15(1): 45–51, Available from: https://www.ncbi.nlm.nih.gov/pmc/articles/PMC4365846/PMC4365846 25829880

[25] Mareninova OA Hermann K French SW , et al. Impaired autophagic flux mediates acinar cell vacuole formation and trypsinogen activation in rodent models of acute pancreatitis. J Clin Investig. 2009; 119(11): 3340–3355. 10.1172/JCI38674 PMC2769194 19805911

[26] Sah RP Saluja AK . Trypsinogen activation in acute and chronic pancreatitis: is it a prerequisite? Gut 2011; 60(10): 1305–1307. 10.1136/gut.2011.241703 PMC3682650 21672938

[27] Gapp J Chandra S Tariq A . Acute pancreatitis. StatPearls Publishing, 2023. Available from: https://www.ncbi.nlm.nih.gov/books/NBK482468/29494075

[28] Pitchumoni CS Agarwal N Jain NK . Systemic complications of acute pancreatitis. Am J Gastroenterol 1988; 83(6): 597–606, Available from: https://pubmed.ncbi.nlm.nih.gov/3287900/3287900

[29] St. John Smith E . Advances in understanding nociception and neuropathic pain. J Neurol 2017; 265(2): 231–238, Available from: https://www.ncbi.nlm.nih.gov/pmc/articles/PMC5808094/10.1007/s00415-017-8641-6 PMC5808094 29032407

[30] Raja SN Carr DB Cohen M , et al. The revised international association for the study of pain definition of pain: concepts, challenges, and compromises. Pain 2020; 161(9). 10.1097/j.pain.0000000000001939 PMC7680716 32694387

[31] Botha C Farmer AD Nilsson M , et al. Preliminary report: modulation of parasympathetic nervous system tone influences oesophageal pain hypersensitivity. Gut 2015; 64(4): 611–617. 10.1136/gutjnl-2013-306698, Available from: https://pubmed.ncbi.nlm.nih.gov/24870622/text/Conclusions/3A/20The/20development/20of/20oesophageal 24870622

[32] Yam M Loh Y Tan C , et al. General pathways of pain sensation and the major neurotransmitters involved in pain regulation. Int J Mol Sci 2018; 19(8): 2164. 10.3390/ijms19082164, Available from: https://www.ncbi.nlm.nih.gov/pmc/articles/PMC6121522/PMC6121522 30042373

[33] Jin Y Liu L Chen B , et al. Involvement of the PI3K/Akt/NF-κB signaling pathway in the attenuation of severe acute pancreatitis-associated acute lung injury by Sedum sarmentosum Bunge extract. BioMed Res Int 2017; 2017: 1–13. 10.1155/2017/9698410 PMC5735615 29359164

[34] Xue M Han L Qian W , et al. Nitric oxide stimulates acute pancreatitis pain via activating the NF-κB signaling pathway and inhibiting the kappa opioid receptor. Oxid Med Cell Longev 2020; 2020: 1–13. 10.1155/2020/9230958 PMC7231422 32454946

[35] Drewes AM Krarup AL Detlefsen S , et al. Pain in chronic pancreatitis: the role of neuropathic pain mechanisms. Gut 2008; 57(11): 1616–1627. 10.1136/gut.2007.146621 18566105

[36] Matsuda M Huh Y Ji RR . Roles of inflammation, neurogenic inflammation, and neuroinflammation in pain. J Anesth 2018; 33(1): 131–139. 10.1007/s00540-018-2579-4 PMC6813778 30448975

[37] Knoph CS PAINAP Collaborative Pandanaboyana S . Pain intensity and prognosis of acute pancreatitis in an international, prospective study. Br J Surg. 2025; 112(8): znaf155. 10.1093/bjs/znaf155 PMC12362069 40827650

[38] Rasmussen PV Sindrup SH Jensen TS , et al. Symptoms and signs in patients with suspected neuropathic pain. Pain 2004; 110(1): 461–469. 10.1016/j.pain.2004.04.034 15275799

[39] Drewes AM Olesen AE Farmer AD , et al. Gastrointestinal pain. Nat Rev Dis Primers 2020; 6(1): 1. 10.1038/s41572-019-0135-7 31907359

[40] Collett B . Visceral pain: the importance of pain management services. Br J Pain 2013; 7(1): 6–7. 10.1177/2049463713480138 PMC4590154 26516492

[41] Meerschaert KA Chiu IM . The gut–brain axis and pain signalling mechanisms in the gastrointestinal tract. Nat Rev Gastroenterol Hepatol 2024; 22(3): 206–221. 10.1038/s41575-024-01017-9. Available from: https://www.nature.com/articles/s41575-024-01017-9/fromPaywallRec/true PMC13737996 39578592

[42] High KN Vera–Portocarrero LP . Visceral pain model, pancreatic pain. Springer eBooks, 2007, pp. 2630–2634.

[43] Osterweis M Kleinman A Mechanic D . The anatomy and physiology of pain. Nih.gov. National Academies Press (US), 2019. Available from: https://www.ncbi.nlm.nih.gov/books/NBK219252/25032476

[44] Al-Leswas D Baxter N Lim WB , et al. The safety and efficacy of epidural anaesthesia in acute pancreatitis: a systematic review and meta-analysis. HPB 2023; 25(2): 162–171. 10.1016/j.hpb.2022.12.004 36593161

[45] Waldman SD Patt RB . Splanchnic and celiac plexus nerve block. Elsevier eBooks, 2011, pp. 1191–1204.

[46] Arcidiacono PGG Calori G Carrara S , et al. Celiac plexus block for pancreatic cancer pain in adults. Cochrane Database Syst Rev. 2011; 2011(3). 10.1002/14651858.CD007519.pub2 PMC6464722 21412903

[47] Fusaroli P Caletti G . Is there a role for celiac plexus block for chronic pancreatitis? Endosc Int Open 2015; 3(1): E60–E62. 10.1055/s-0034-1391392, Available from: https://www.ncbi.nlm.nih.gov/pmc/articles/PMC4423247/PMC4423247 26134774

[48] Procacci P Maresca M . Referred pain from somatic and visceral structures. Curr Rev Pain 1999; 3(2): 96–99. 10.1007/s11916-999-0032-y

[49] Brumovsky PR Gebhart GF . Visceral organ cross-sensitization – an integrated perspective. Auton Neurosci 2010; 153(1–2): 106–115. 10.1016/j.autneu.2009.07.006 PMC2818077 19679518

[50] Borowczyk J. Visceral referred pain. Encyclopedia of Pain, 2020, vol 2645–2647. Available from: 10.1007/2F978-3-540-29805-2_4801/text/Definition

[51] Lieb IIJG Forsmark CE . Review article: pain and chronic pancreatitis. Aliment Pharmacol Ther 2009; 29(7): 706–719. 10.1111/j.1365-2036.2009.03931.x 19284407

[52] Bohren Y Daniel IT Muller A , et al. Platelet-rich plasma and cytokines in neuropathic pain: a narrative review and a clinical perspective. Eur J Pain 2021; 26(1): 43–60. 10.1002/ejp.1846 34288258

[53] Finnerup NB Haroutounian S Kamerman P , et al. Neuropathic pain: an updated grading system for research and clinical practice. Pain 2016; 157(8): 1599–1606. 10.1097/j.pain.0000000000000492, Available from: https://www.ncbi.nlm.nih.gov/pmc/articles/PMC4949003/PMC4949003 27115670

[54] Fitzcharles MA Cohen SP Clauw DJ , et al. Nociplastic pain: towards an understanding of prevalent pain conditions. Lancet 2021; 397(10289): 2098–2110. 10.1016/s0140-6736(21)00392-5, Available from: https://www.thelancet.com/pdfs/journals/lancet/PIIS0140-6736(21)00392-5.pdf 34062144

[55] Tuck NL Teo K Kuhlmann L , et al. Pain patterns in chronic pancreatitis and chronic primary pain. Pancreatology 2022; 22(5): 572–582. 10.1016/j.pan.2022.04.016, Available from: https://www.sciencedirect.com/science/article/abs/pii/S1424390322001673 35562269

[56] van Zeggeren L Boelens Nabbi R Kallewaard JW , et al. 16. Pain in chronic pancreatitis. Pain Pract 2025; 25: e70030. 10.1111/papr.70030 PMC11973027 40189721

